# The Truth about Homœopathy

**Published:** 1895-03

**Authors:** 


THE TRUTH ABOUT HOMŒOPATHY. By Dr. Wm. H. Holcombe. A posthumous manuscript, also a sketch of the life of Dr. Holcombe. Philadelphia: Bœricke & Tafel. 1894. Price, 25 cents. Cloth.

This little book is an answer to the Prize Essay against Homœopathy by Dr. W. W. Browning, of Brooklyn, N. Y., who wrote it to win the prize of $100 offered by Dr. Geo. M. Gould, of Philadelphia, editor of *The Medical News.* The profession will remember the stir made in the professional circles of both schools by the attacks of Dr. Gould a couple of years ago.

The essay was the last manuscript ever penned by Dr. Holcombe, and was found among his papers after his death.

It is written in his usual fine style for which he was justly famous, but betrays here and there his own objections to "higher dilutions."

In its answers to each specific charge of Dr. Browning it is masterly. His exceedingly great familiarity with the history of Homœopathy, and his accurate memory for the facts accumulated by the lapse of time, are well shown in this little pamphlet.

